# RNA-seq reveals differentially expressed genes in rice (*Oryza sativa*) roots during interactions with plant-growth promoting bacteria, *Azospirillum brasilense*

**DOI:** 10.1371/journal.pone.0217309

**Published:** 2019-05-23

**Authors:** Jacklyn Thomas, Ha Ram Kim, Yasir Rahmatallah, Grant Wiggins, Qinqing Yang, Raj Singh, Galina Glazko, Arijit Mukherjee

**Affiliations:** 1 Department of Biology, University of Central Arkansas, Conway, Arkansas, United States of America; 2 Department of Biomedical Informatics, University of Arkansas for Medical Sciences, Little Rock, Arkansas, United States of America; Institute of Genetics and Developmental Biology Chinese Academy of Sciences, CHINA

## Abstract

Major non-legume crops can form beneficial associations with nitrogen-fixing bacteria like *Azospirillum brasilense*. Our current understanding of the molecular aspects and signaling that occur between important crops like rice and these nitrogen-fixing bacteria is limited. In this study, we used an experimental system where the bacteria could colonize the plant roots and promote plant growth in wild type rice and symbiotic mutants (*dmi3* and *pollux*) in rice. Our data suggest that plant growth promotion and root penetration is not dependent on these genes. We then used this colonization model to identify regulation of gene expression at two different time points during this interaction: at 1day post inoculation (dpi), we identified 1622 differentially expressed genes (DEGs) in rice roots, and at 14dpi, we identified 1995 DEGs. We performed a comprehensive data mining to classify the DEGs into the categories of transcription factors (TFs), protein kinases (PKs), and transporters (TRs). Several of these DEGs encode proteins that are involved in the flavonoid biosynthetic pathway, defense, and hormone signaling pathways. We identified genes that are involved in nitrate and sugar transport and are also implicated to play a role in other plant-microbe interactions. Overall, findings from this study will serve as an excellent resource to characterize the host genetic pathway controlling the interactions between non-legumes and beneficial bacteria which can have long-term implications towards sustainably improving agriculture.

## Introduction

Plants can form beneficial mutualistic associations with a diverse array of microbes including soil bacteria rhizobia, arbuscular mycorrhizal fungi (AMF), plant-growth promoting bacteria (PGPB), etc. [1–3]. Among these associations, the legume-rhizobia symbiosis is the most studied and efficient symbiosis. It occurs between plants from the legume family (pea, soybean, beans, etc.) and rhizobia culminating in the development of root nodules inside which the rhizobia fix atmospheric nitrogen for the host plant in exchange for carbohydrates [2, 3]. Decades of genetic and biochemical studies have identified the plant and microbial signals controlling the establishment of this symbiosis [2, 3]. Genetic studies in legumes also identified several plant genes involved at different stages (from initiation to regulation) of this symbiosis [2, 3]. Some of the genes required in the initial stages include a cation channel (*DMI1/POLLUX* and *CASTOR*), a nuclear calcium and calmodulin-dependent kinase (*DMI3/CCaMK*), a substrate of *DMI3* (*IPD3/CYCLOPS*), and a receptor-like kinase (*DMI2/SYMRK*) among others [2, 3]. Later studies showed that some of these genes are also required for the establishment of symbiosis with arbuscular mycorrhizal fungi leading to the concept of the common symbiotic pathway (CSP) [2–4]. Some genes from the CSP have also been shown to be required in actinorhizal symbiosis and non-symbiotic interactions [4]. The large body of elegant genetic studies in legumes has significantly improved our understanding of the host genetic pathways controlling legume-rhizobia symbiosis. Unfortunately, the same depth of information does not exist for other beneficial plant-microbe interactions such as the ones occurring between non-legumes and plant-growth promoting bacteria.

Majority of non-legume crops benefit from interactions with plant-growth promoting bacteria. Several studies have shown that the biological nitrogen fixation (BNF) in non-legumes comes from diazotrophic (nitrogen-fixing) bacteria in several genera of alpha- and beta-proteobacteria including *Azospirillum*, *Azorhizobium*, *Herbaspirillum*, *Burkholderia*, etc. [1]. Unlike legume-rhizobia endosymbiosis, these bacteria induce no specialized root structures and are different in their colonization characteristics. *Azospirillum brasilense* represents the best-characterized genus of plant growth-promoting bacteria with a diverse host range including important cereals. These promote plant growth by several mechanisms including nitrogen fixation and phytohormone secretion [5]. *A*. *brasilense* has emerged as a great model for studying nitrogen-fixing bacteria with its sequenced genome and feasibility to genetic manipulation like transposon mutagenesis [5, 6]. However, unlike legume-rhizobia symbiosis, there are still only limited data available on the molecular aspects and signaling in the interactions between non-legumes like rice and diazotrophic bacteria [1, 7, 8].

In this study, we set up an experimental system in which the *A*. *brasilense* Sp245 strain could colonize rice roots and promote growth under controlled, sterile conditions. We also studied if *A*. *brasilense* could promote plant growth and penetrate the roots of symbiotic mutants in rice. To identify the plant genes and pathways involved during rice-*A*. *brasilense* interactions, we performed transcriptional profiling by RNA-seq. This study provides an excellent resource to further our understanding of the molecular mechanisms occurring in rice roots during its interaction with *A*. *brasilense*.

## Results

### *Azospirillum brasilense* promoted rice growth under controlled experimental conditions

**Fig 1:**
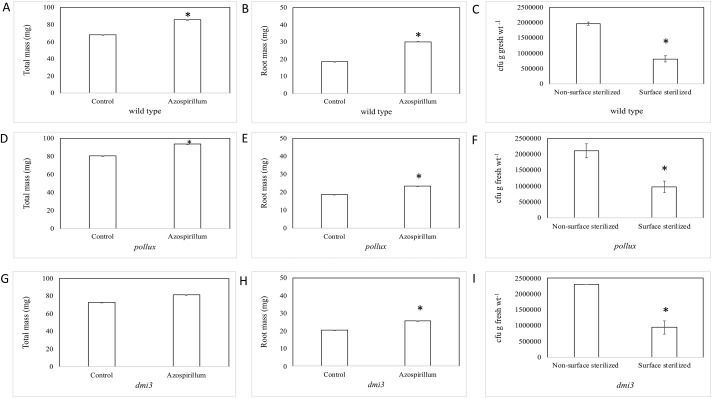
Growth promotion and root colonization in wild-type rice and symbiotic mutants by *A*. *brasilense*. (A, D, G) show that total plant mass (mg) increased in wild-type rice, *pollux*, and *dmi3* mutants upon inoculation with *A*. *brasilense*. Data represents the average of five experimental replications (n = 10–15) +/- SE. Asterisk (*) denotes significance between the conditions by *t*-test (*P* < 0.001, *P* < 0.001, *P* < 0.06). (B, E, H) show that root mass (mg) increased in wild-type rice, *pollux*, and *dmi3* mutants upon inoculation with *A*. *brasilense*. Data represents the average of five experimental replications (n = 10–15) +/- SE. Asterisk (*) denotes significance between the conditions by *t*-test (*P* < 0.003, *P* < 0.01, *P* < 0.007). (C, F, I) show comparison of total colony-forming units (cfu) of *A*. *brasilense* determined by serial dilution and plate counts of bacteria between non-surface sterilized and surface sterilized roots of wild-type, *pollux*, and *dmi3* rice seedlings inoculated with *A*. *brasilense*. The data are average of five experiments. Each experiment had at least three plants. Asterisk (*) denotes significance between the conditions by *t*-test (*P* < 0.001, *P* < 0.001, *P* < 0.001).

We investigated if *A*. *brasilense* could promote rice growth under controlled experimental conditions. Our results show that the total plant mass was 1.26-fold higher in *A*. *brasilense*-inoculated wild-type (*Oryza sativa* cv. Nipponbare) rice plants than the uninoculated ones (Fig 1A). Root mass was 1.63-fold higher in the bacteria-inoculated plants than the controls (Fig 1B). Next, we were interested in determining if the bacteria could colonize the plant roots under the same conditions. We used plate count assays and recovered *A*. *brasilense* from surface sterilized rice roots indicating that bacteria could penetrate the roots under these conditions. As expected the number of colonies recovered from the surface sterilized roots was significantly lower (0.41-fold) than the non-surface sterilized roots (Fig 1C).

### *A*. *brasilense* promoted plant growth in rice symbiotic mutants

We investigated if *A*. *brasilense* could promote plant growth in symbiotic rice mutants (*Os-dmi3* and *Os-pollux*) under these controlled experimental conditions. Our results indicate that the total plant mass increased in *A*. *brasilense*-inoculated *Os-pollux* (1.16-fold) and *Os-dmi3* (1.12-fold) plants compared to the uninoculated plants (Fig 1D and 1G). The root mass in *Os-pollux* (1.23-fold) and *Os-dmi3* (1.27-fold) plants also increased upon *A*. *brasilense* inoculation (Fig 1E and 1H). Next, we performed plate count assays and recovered *A*. *brasilense* from surface sterilized roots of *Os-pollux* and *Os-dmi3* indicating that bacteria could penetrate the roots of these rice symbiotic mutants. As observed in wild-type roots, the number of bacterial colonies recovered from the surface-sterilized *pollux* roots was 0.46-fold lower than the non-surface sterilized *pollux* roots (Fig 1F). Similarly, the number of bacterial colonies recovered from the surface-sterilized *dmi3* roots was 0.41-fold lower than the non-surface sterilized *dmi3* roots (Fig 1I).

### Analysis of rice root transcriptome upon inoculation with *A*. *brasilense*

**Fig 2:**
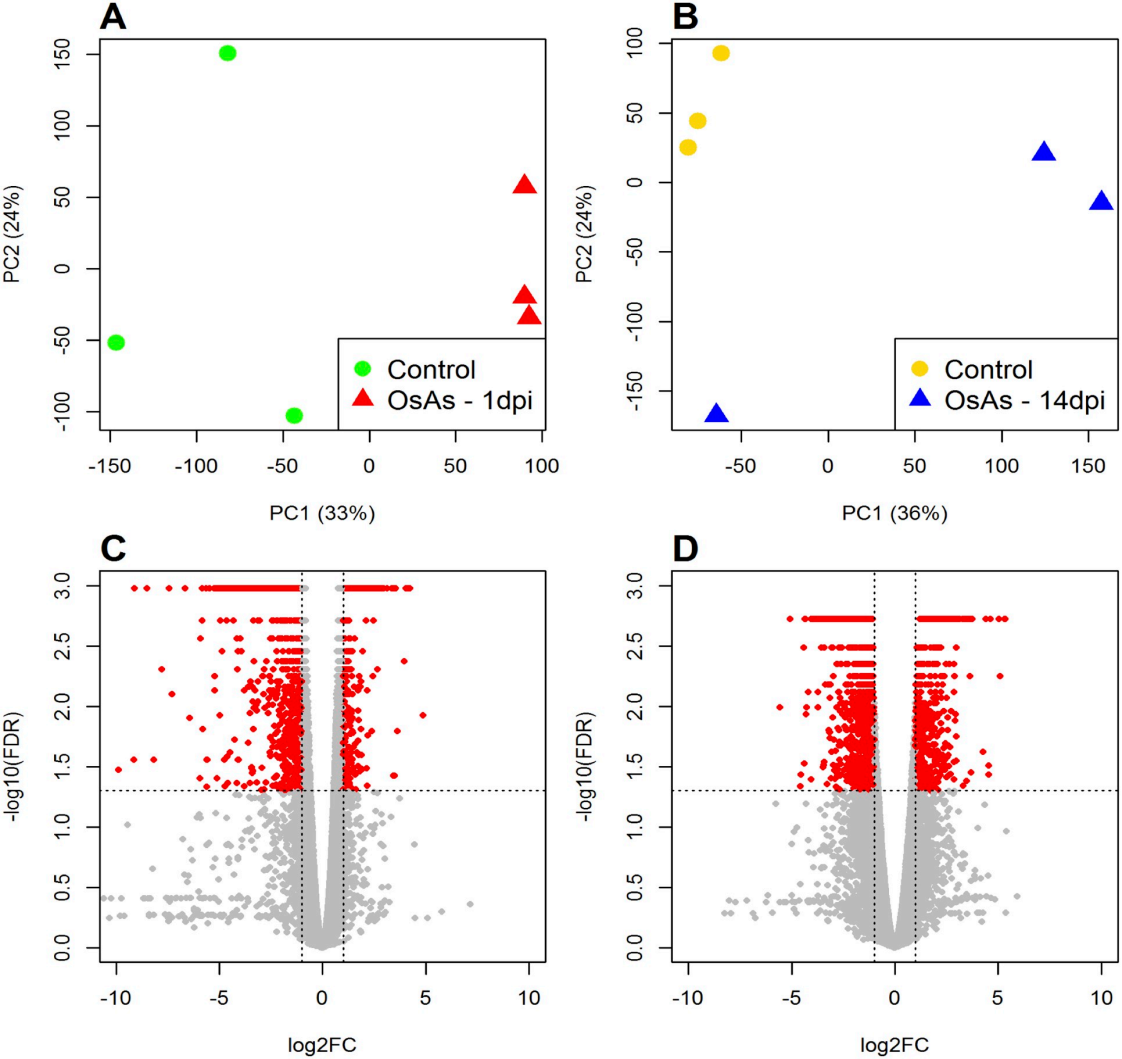
Summary plots for the gene expression profiles and differential expression analysis. Panels A and B show scatter plots of the first two principal components of the FPKM normalized gene expression profiles at 1dpi and 14dpi, respectively. Panels C and D respectively show volcano plots at 1dpi and 14dpi where mean log_2_ fold change is plotted against the –log_10_ FDR adjusted P-values for all expressed genes. Significant DE genes (FDR<0.05 and |FC|>2) were highlighted with red color.

We used high-throughput RNA-sequencing to identify differentially expressed genes (DEGs) in rice roots upon inoculation with *A*. *brasilense*. We analyzed the expression profile of wild-type rice (*Oryza sativa* cv. Nipponbare) in the following experimental groups: (1) 1day post inoculation (dpi): wild-type roots + mock treatment (water only) vs. wild-type roots + *A*. *brasilense*, and (2) 14dpi: wild-type roots + mock treatment (water only) vs. wild-type roots + *A*. *brasilense*. Each treatment group had three biological replicates. Sequencing libraries were prepared from these RNA samples. The completed libraries were quality checked and quantified before sequencing in a 2×150bp paired-end format using HiSeq 4000. An average of 36 million reads was obtained per sample with an average mapping rate of 85% to the rice genome (MSU, version 7) (S1 Table). A good degree of correlation was observed between the biological replicates of each sample (Fig 2A and 2B). To identify the differentially expressed genes (DEGs) from the dataset, an FDR adjusted *P*-value of <0.05 was set and a fold change of >2 (|Log_2_FC| >1) was assigned. At 1dpi and 14dpi, we identified 1622 and 1995 DEGs in rice roots, respectively (Fig 2C and 2D; S2 and S3 Tables). Among these, 300 genes were differentially expressed at both time points. At 1dpi, 490 genes were upregulated in expression, and at 14 dpi, 619 genes were upregulated in expression (S2 and S3 Tables).

**Fig 3:**
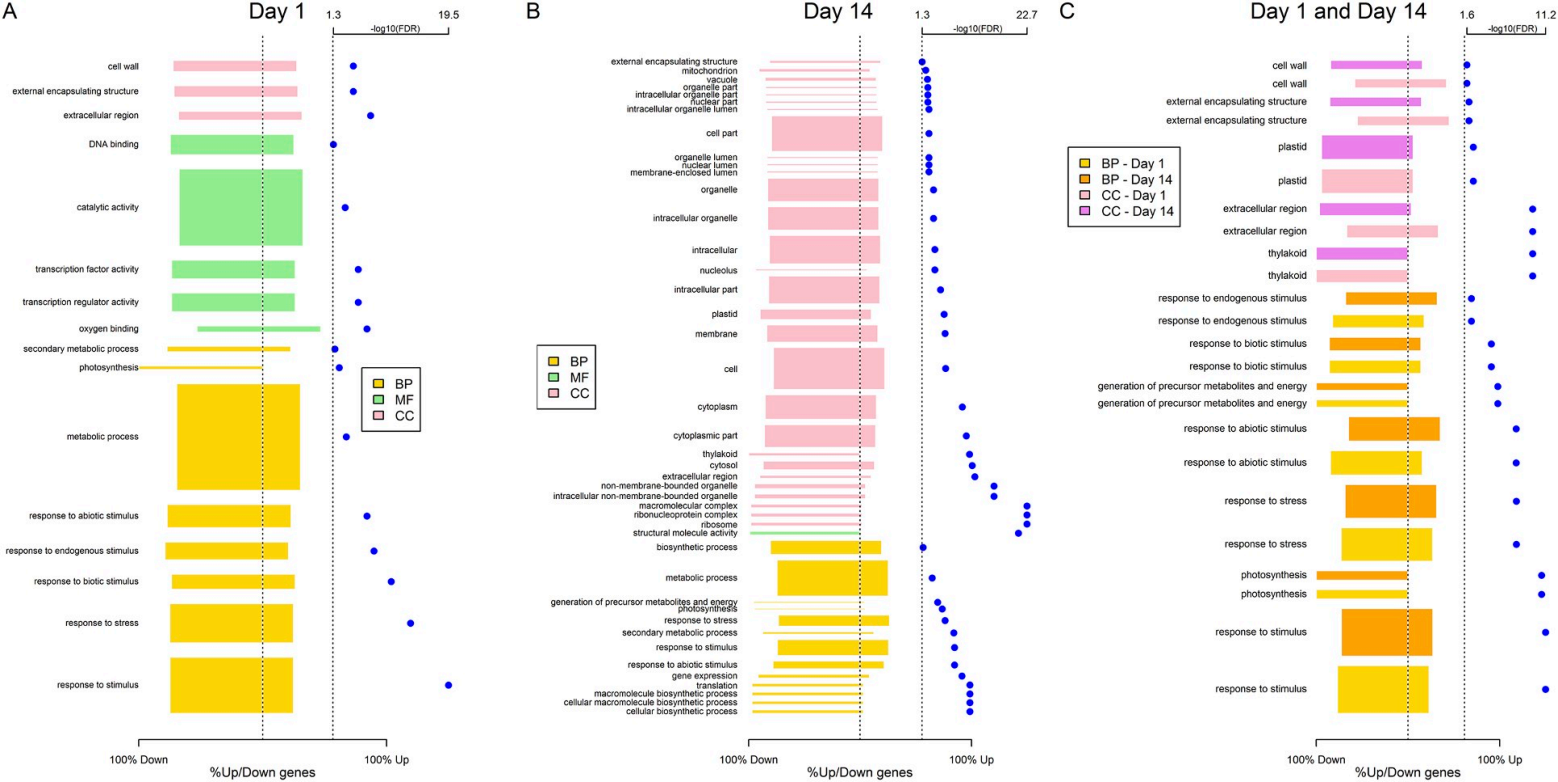
Bar plots summarizing the gene ontology terms over-represented in DE genes between control and *A*. *brasilense* samples for (A) 1dpi (B) 14dpi (C) common in both 1dpi and 14dpi datasets. The magnitude of bars in the positive and negative directions respectively represents the proportion of up-regulated and down-regulated genes associated with each GO term. The width of each bar is proportional to the number of DE genes associated with the GO term. Blue dots represent –log_10_(FDR) for each significant GO term. A vertical dotted line at –log_10_(0.05) = 1.3 indicates the significance threshold. Bar color indicates the three categories of GO term: Biological Processes (BP), Molecular Functions (MF), and Cellular Components (CC).

We performed a gene ontology (GO) analysis to determine the biological significance of the DEGs with respect to biological processes (BP), molecular functions (MF), and cellular localization (CC) of their proteins. We used singular enrichment analysis (SEA) with agriGO [9] and identified 16 GO terms that were significantly enriched at 1dpi. These included 8 in biological processes (e.g., response to stimulus, response to biotic stimulus, metabolic process, etc.), 5 in molecular functions (e.g., transcription factor activity, catalytic activity, etc.) and 3 in the cellular component (e.g., cell wall, extracellular region, etc.) (Fig 3A). At 14dpi, we identified 43 GO terms that were significantly enriched. These included 13 in biological processes (e.g., response to stimulus, gene expression, etc.), 1 in molecular function (structural molecule activity), and 29 in cellular components (e.g., membrane, cytosol, etc.) (Fig 3B). In the 300 genes that were differentially expressed at both time points, we identified 12 significantly enriched GO terms including 7 in biological processes (e.g., response to stimulus, response to endogenous stimulus, etc.) and 5 in cellular components (e.g., extracellular region, cell wall, etc.) (Fig 3C).

### Comprehensive data mining of the transcriptome dataset

Several studies have shown that transcription factors (TFs), protein kinases (PKs), and transporters (TRs) play critical roles in signal transduction pathways involved in important biological processes including plant-microbe interactions [10–15]. Genes belonging to the flavonoid synthetic pathway, hormone signaling and plant defense have also been shown to be involved in plant-microbe interactions [16–23]. As such the next logical step was to perform a comprehensive data mining and identify the genes in these different categories. We report selected DEGs from these gene classes identified in our dataset in the next sections.

We identified 146 and 85 differentially expressed transcription factors at 1 and 14dpi, respectively (S4 Table). Many of these belong to major plant TF families such as AP2/ERF (APETALA 2/Ethylene response factor) family, MYB (myeloblastosis oncogene) family, WRKY family, NAC (NAM, ATAF1/2, and CUC) domain, and the GRAS (GAI, RGA, and SCR) family. At both time points, the major TFs that were upregulated in expression were NAC domain-containing proteins, AP2/ERFs, and MYB family of TFs among others. For instance, six TFs belonging to the AP2/ERF family were differentially expressed across both time points (S4 Table).

We identified 110 protein kinases that were differentially expressed at 1dpi and 109 PKs that were differentially expressed at 14dpi (S5 Table). Some of the major PKs well represented in our dataset included the CAMK (calcium/calmodulin-dependent kinases), SHR5 receptor-like kinases, and OsWAK receptor-like kinases among others. We also identified two peptidoglycan-binding LysM domain-containing protein at each time point. Additionally, we identified 16 PKs that were differentially expressed at both time points (S5 Table).

We identified 106 differentially expressed transporters at 1dpi, and 124 differentially expressed transporters at 14dpi (S6 Table). Major transporters identified were nitrate transporters, ammonium transporters, sugar transporters, peptide transporters, ABC-2 type transporters, and several nodulins (*MtN3*, *Major facilitator superfamily*, etc.). We also identified three differentially expressed auxin efflux carriers: one at 1dpi and two at 14dpi. Nineteen transporters were differentially expressed at both time points including two nitrate transporters, a sugar transporter, and some nodulin genes (S6 Table).

In our dataset, we identified several genes belonging to the flavonoid biosynthetic pathway that were differentially expressed. These included chalcone synthase genes, chalcone-flavonone isomerase genes, flavonol synthase genes, and naringenin synthesis genes (S2 and S3 Tables). Many hormone-related genes were differentially expressed in the dataset. These were auxin efflux carriers, auxin-responsive genes, auxin response factors, 1-aminocyclopropane-1carboxylate (ACC) oxidase genes, ethylene insensitive 2 (EIN2) gene, cytokinin-O-glucosyltransferases, and cytokinin dehydrogenase precursors among others (S2 and S3 Tables). Several defense-related genes were also differentially regulated in expression. Some of these were pathogenesis-related genes, chitinases, thionin genes, and cinnamoyl-CoA-reductases (S2 and S3 Tables).

### Gene expression validation

**Fig 4:**
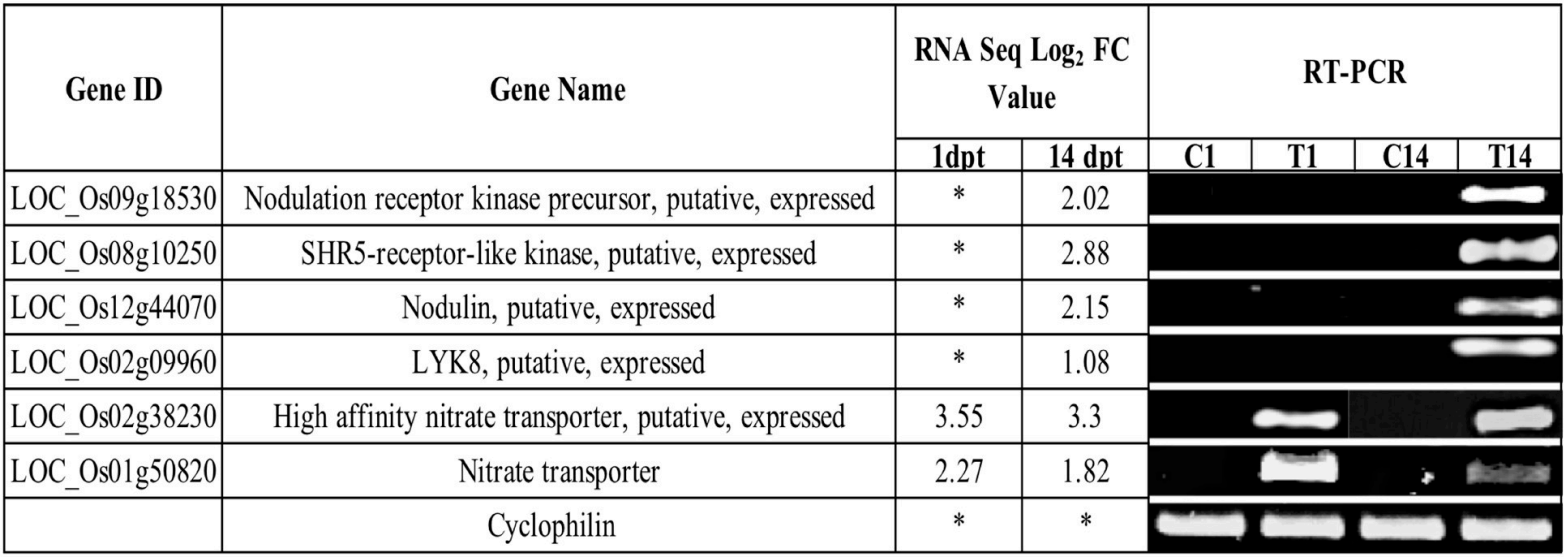
RT-PCR validation of differentially expressed genes identified by RNA-Seq. Expression pattern of six DEGs was validated by RT-PCR. For the RT-PCR experiments, C1 and T1, C14 and T14 represent cDNA templates synthesized from control and treatment RNA samples at 1- and 14dpi respectively. RT-PCR was performed in at least three biological replicates for all the samples. *Cyclophilin* was used as an internal reference gene.

To validate the gene expression patterns identified in our RNA-Seq dataset, we performed reverse transcription polymerase chain reaction (RT-PCR) for six genes (Fig 4). Primers designed for RT-PCR were based on the Rice Genome Annotation Project database annotations, and the primer sequences are listed in S7 Table. Overall, the RT-PCR results confirm the expression pattern of these genes identified in the RNA-seq experiment (Fig 4).

## Discussion

Non-legume crops such as rice, maize, and wheat can benefit from associations with plant-growth promoting bacteria. These bacteria promote plant growth by several mechanisms including nitrogen fixation and phytohormone secretion [5]. Although several studies have looked into the colonization mechanisms by which different nitrogen-fixing bacteria penetrate plant roots, not much is known about the molecular mechanisms controlling these associations. In this study, we established an experimental system in which *Azospirillum brasilense* could colonize rice roots under sterile, controlled conditions and promote plant growth. Interestingly, *A*. *brasilense* promoted growth in two rice symbiotic mutants (*pollux* and *dmi3*). The *POLLUX* and *DMI3* genes belong to a very well-characterized pathway in plant-microbe symbioses known as the common symbiotic pathway (CSP) [4]. Genes belonging to the CSP are required for the establishment of the two major plant-microbe endosymbioses: legume-rhizobia symbiosis and arbuscular mycorrhizal symbiosis. Besides these symbioses, the actinorhizal symbiosis also requires genes from the CSP [4]. To the best of our knowledge, not much is known about this pathway’s role in interactions between plants and plant growth promoting bacteria like *A*. *brasilense*. Here we show that *A*. *brasilense* can promote plant growth independent of the CSP and can penetrate the roots of these symbiotic mutants. However, further studies need to be conducted to understand the role of this pathway during interactions between nitrogen-fixing bacteria and their host plants. Our results also suggest that the host plant probably uses other genetic pathway(s) to accommodate the microbe.

In this study, we performed an RNA-sequencing experiment to identify the regulation of gene expression occurring in rice roots during interactions with *A*. *brasilense* at two different time points (1- and 14dpi). We identified hundreds of differentially expressed genes in rice roots at both time points. We hypothesize that transcription factors, protein kinases, and transporters are likely going to be involved in the host genetic pathway controlling the interaction between rice and *A*. *brasilense*. We also hypothesized that hormone-related genes and defense genes would be differentially regulated during this interaction. So, we performed a comprehensive data mining of the RNA-seq dataset and identified these classes of genes which would be excellent targets to characterize the host genetic pathway controlling this important plant-microbe association. Below we discuss some selected genes from our dataset based on their role in other plant-microbe associations such as the legume-rhizobia symbiosis (LRS) and arbuscular mycorrhizal (AM) symbiosis.

### Flavonoid biosynthetic pathway

Flavonoids are essential signals required for the initiation and establishment of legume-rhizobia symbiosis. They are also key regulators of other root endosymbioses such as AM and actinorhizal symbioses [16, 24]. Several studies have also reported that flavonoids may be involved in other plant-microbe interactions [16]. For instance, some flavonoids were found to stimulate colonization of wheat by *A*. *brasilense* and *Azorhizobium caulinodans* [25]. In our study, several genes involved in the flavonoid biosynthetic pathway were differentially expressed in rice roots. At 1dpi, one chalcone synthase gene (LOC_Os10g08670) was upregulated in expression while another (LOC_Os07g34260) was downregulated in expression in rice roots. At 14dpi, we identified three chalcone synthase genes (LOC_Os07g34260, LOC_Os11g32650, and LOC_Os07g31770) and two chalcone-flavonone isomerase genes (LOC_Os11g02440 and LOC_Os12g02370) that were downregulated in expression. Also, at 1dpi we identified three flavonol synthase genes (LOC_Os01g61610, LOC_Os03g03034, and LOC_Os02g52840) and two naringenin synthesis genes (LOC_Os04g49194 and LOC_Os04g56700) that were differentially expressed. Interestingly, none of these genes were differentially expressed at 14dpi suggesting a different role of these genes and subsequently these flavonoids at the later time point. It will be interesting to determine if the expression pattern of these plant genes is correlated with communication with its microbial partner and eventual accommodation of the microbe.

### Defense-related genes

During interactions with beneficial microbes, the host plant adjusts its defense mechanisms accordingly to facilitate the interaction. Several articles on symbioses have reported suppression of defense-related gene expression in the host plant [17–19, 26]. In our dataset, we observed several well-characterized plant defense-related genes to be downregulated in expression. In general, accumulation of chitinases has been associated with defense against pathogens [27, 28]. Here we identified five chitinase genes (LOC_Os02g39330, LOC_Os03g30470, LOC_Os04g41680, LOC_Os05g33140, and LOC_Os04g41620) at 1dpi and four chitinase genes (LOC_Os09g32080, LOC_Os10g39680, LOC_Os03g04060, and LOC_Os05g33140) at 14dpi that were downregulated in expression. Chitinases have been implicated with root nodulation and even protect nodules against pathogens [29–33]. Another set of genes, the pathogenesis-related (PR) genes, associated with plant defense was observed to be downregulated in expression in rice roots. The PR genes (LOC_Os12g36830, LOC_Os12g36840, LOC_Os12g36880, LOC_Os04g50700, and LOC_Os04g50700) were all downregulated in expression at 14dpi. Interestingly, only one of these genes (LOC_Os12g36880) was downregulated in expression at 1dpi. Several of these PR genes are strongly induced in rice upon inoculation with the pathogen, *Magnaporthe oryzae*, and are considered to be excellent markers for plant defense reactions [34]. Another study reported that the PR gene (LOC_ Os12g36840) was suppressed in expression in rice during interactions with the plant-growth promoting bacteria, *Herbaspirillum seropedicae* [8]. We also observed several thionin genes to be downregulated in expression at 1dpi (e.g., LOC_Os03g14300, LOC_Os06g31280, LOC_Os06g31800) and 14dpi (e.g., LOC_Os06g32020, LOC_Os06g31280, LOC_Os06g31890, LOC_Os11g15250). Interestingly, some thionin genes were upregulated in expression at both time points. A thionin gene was also found to be differentially expressed in rice roots during interactions with *Herbaspirillum* [8]. Several studies have shown that cinnamoyl-CoA-reductase, a key enzyme in lignin biosynthesis, plays a role in defense-related processes in rice [35–37]. One study showed that expression of a cinnamoyl-CoA-reductase (LOC_Os08g34280) was induced during interactions with a pathogenic microbe but repressed during interactions with a mutualistic microbe [36]. In our study, the expression of this gene was downregulated at 14dpi. Also, we identified another cinnamoyl-CoA-reductase gene (LOC_Os02g56700) that was downregulated in expression at both time points. Collectively these expression data suggest that during rice-*A*. *brasilense* interactions, the plant is reprogramming its defense-related genes similar to other interactions between plants and beneficial microbes.

### Transporters

Nitrate transporters have been shown to transport not only nitrate but other substrates, including peptides, amino acids, and plant hormones such as auxin and have been involved in processes from nitrogen sensing to nitrogen use efficiency [13]. Studies in *L*. *japonicus* and *M*. *truncatula* have shown that nitrate transporters play key roles in nitrate signaling, root growth, and nodulation [12]. Since *A*. *brasilense* can stimulate plant growth via improved nitrogen uptake [38], this class of transporters is likely to play essential roles in this plant-microbe interaction. Plants have evolved two nitrate uptake systems to adapt to nitrate availability: a low-affinity transporter system and a high-affinity transporter system [13, 39]. In our study, we identified both high-affinity transporters and low-affinity nitrate transporters (e.g., peptide transporter family). The high-affinity nitrate transporters (LOC_Os02g38230, LOC_Os01g50820) were upregulated in expression at both time points suggesting that these are likely involved at all stages of this interaction. We identified several low-affinity nitrate transporters like the peptide transporters (e.g., LOC_Os10g02080, LOC_Os03g04570, LOC_Os01g65130, LOC_Os01g65140) to be differentially expressed in rice roots. A recent study showed that a peptide transporter contributed to nitrogen allocation and increased grain yield in rice [40]. Besides nitrate, another form of nitrogen available to plants is ammonium. Ammonium transporters are important for high-affinity primary uptake and translocation of ammonium in plants. These transporters have been shown to play crucial roles in beneficial plant-microbe symbioses: legume-rhizobia symbiosis and AM symbiosis [41]. In this study, we identified three differentially expressed ammonium transporters (LOC_Os02g40710, LOC_Os02g40730, and LOC_Os04g43070) in rice roots. In plant-microbe symbioses, the host plant benefits from improved nutrient uptake in exchange for carbohydrates to its symbiotic partner. Studies in legume-rhizobia symbiosis have shown that sucrose transport is essential for symbiotic nitrogen fixation because of the expensive nature of the process. Sucrose transporters were shown to play an active role in the loading and unloading of sugar in the phloem, transfer of sugar to the nodules and subsequently to bacteria within nodules [42]. Additionally, these transporters were also induced during mycorrhization, which suggests that they may also play an important role in sugar efflux to fungal symbionts [43, 44]. We identified several sugar transporters that were differentially expressed in rice roots. One sugar transporter (LOC_Os04g37970) was upregulated in expression at both time points suggesting a possible role in this rice-*A*. *brasilense* interaction. Another major group of transporters identified in our dataset includes the nodulin (*MtN3*, *Major facilitator superfamily*, etc.) genes. Interestingly, nodulin genes were first characterized in the initial response during the development of symbiotic root nodules and considered legume-specific. Recent studies have identified these genes to be present in non-legumes and have been suggested to play key roles in hormone and solute transport during other processes [45]. Future studies can investigate the role(s) of these genes in other plant-microbe associations in non-legumes.

### Receptor kinases

Plant receptor-like kinases (RLKs) play vital roles in diverse signaling pathways that are involved in plant growth and development, plant defense responses, and plant-microbe symbiosis. Some are also involved in the perception of microbial signaling molecules which is vital to both disease resistance and symbiosis. For instance, the Lysin motif receptor-like kinases (LysM-RLKs) can control the establishment of AM symbiosis and legume-rhizobia symbiosis by recognizing the fungal and bacterial signaling molecules [46, 47]. While most studies on these genes have been performed on legumes, recent studies show that LysM-RLK proteins with an active kinase domain (LYKs) regulate symbiosis in non-legume plants as well [46, 48, 49]. However, not much is known about the role of these genes beyond legume-rhizobia symbiosis and AM symbiosis. In this study, the rice ortholog of *LjNFR5*/*MtNFP* gene (LOC_Os03g13080) was upregulated in expression at 1dpi. We also identified the *LYK8* gene (LOC_Os02g09960) to be upregulated in expression at 14dpi. Future studies should clarify the role of these genes in rice-*A*. *brasilense* interactions. In our study, we identified several SHR5 RLKs that were differentially expressed at both time points. This class of RLKs is present in a wide range of plant species. One study showed that expression of *SHR5* gene was down-regulated in sugarcane plants associated exclusively with beneficial endophytic bacteria [50]. In our study, we identified both upregulated and downregulated *SHR5* genes at both time points. For instance, the *SHR5* gene (LOC_Os05g16430) was upregulated in expression at both time points whereas, the *SHR5* gene (LOC_Os08g10310) was downregulated in expression at both time points. AGC protein kinases are another important family of proteins that seem to regulate the interaction with diverse microbes including both pathogenic and symbiotic microbes [51–53]. In our dataset, we identified one AGC kinase (LOC_Os09g31210) that was up-regulated in expression at 1dpi but downregulated in expression at 14dpi. Another AGC kinase (LOC_Os12g01140) was upregulated in expression in rice roots at 14dpi. The role of these genes in rice-*A*. *brasilense* interactions needs additional investigation.

### Transcription factors

Transcription factors are important regulators of various plant processes from growth and development to beneficial plant-microbe interactions [10, 11]. Genetic studies have identified several transcription factors that are involved in legume-rhizobia symbiosis and AM symbiosis. One example is the AP2/ERF class of TFs which is one of the largest families of plant transcription factors [54]. Multiple studies in *M*. *truncatula* and *L*. *japonicus* have identified different AP2/ERFs that are required at various stages of root nodulation [55–57]. In this study, we identified several genes in this category that were differentially expressed in rice roots. Some of these AP2/ERFs (e.g., LOC_Os4g57340, LOC_Os05g29810, LOC_Os04g52090, and LOC_Os02g42585) were differentially expressed at both time points. Functional characterization of these genes will provide more insights into their role during interactions between rice and *A*. *brasilense*. Another important class of TFs that is exclusive to plants and have been involved in diverse processes including the GRAS family of TFs. Genetic studies have shown that these TFs are required during beneficial plant-microbe symbioses [11, 58, 59]. We identified one GRAS TF (LOC_Os11g47920) that was upregulated in expression at 14dpi and two genes (LOC_Os12g04200 and LOC_Os11g47890) that were downregulated in expression at 1dpi. NAC transcription factors are one of the largest families of plant TFs that have been shown to play important roles in plant-biotic interactions [60]. We identified several genes belonging to the NAC TF family to be differentially expressed at both time points. Interestingly, several of these genes (e.g., LOC_Os10g42130, LOC_Os04g52810, and LOC_Os10g33760) were upregulated at 1dpi suggesting a role at earlier stages. This group of TFs is essential for hormone signaling and plant development including lateral root formation and root development [61–63]. One study showed that a NAC TF was upregulated in expression in central symbiotic nodule tissues in *M*. *truncatula* [62]. Studies have shown that hormone-related TFs are involved in plant-microbe interactions. In addition to the different ethylene response factors, we identified auxin response factors (ARF) to be differentially expressed in our dataset. These ARFs are likely to bind to target genes and regulate them transcriptionally which will induce appropriate physiological responses in a tissue-specific manner. In this study, *ARF11* (LOC_Os04g56850) and *ARF5* (LOC_Os02g04810) were downregulated in expression in rice roots 1dpi with *A*. *brasilense*. One recent study in *M*. *truncatula* showed that changes in expression of auxin response factors occurred during the response to *Sinorhizobium meliloti* infection suggesting a possible role of this family of TFs in nodulation [64]. Interestingly, *MtARF5* and *MtARF11* expression were reduced in *Medicago* roots, similar to what we observed in this study. Future studies can focus on profiling the expression patterns of the ARFs in different plant tissues during *A*. *brasilense* infection.

### Hormone-related genes

Phytohormones play critical regulatory roles in plant growth and development and plant-microbe interactions [2, 20–23]. Plant hormones have their intricate systems requiring protein kinases, transporters, and transcription factors, some of which we discussed in the earlier sections. In this section, we focus on a few hormone-related genes that were identified in our dataset. Auxin is probably the most-studied plant hormone because of its central role in several plant developmental processes. It also plays a crucial role during beneficial plant-microbe symbioses [65–67]. In our dataset, we identified several auxin-related genes including the auxin efflux carriers and auxin-responsive genes among others. Auxin efflux carriers are involved during root nodulation [68]. Here at 1dpi, we identified one auxin efflux carrier gene (LOC_Os01g45550) that was downregulated in expression. At 14dpi, we identified two auxin efflux carriers (LOC_Os01g58860, LOC_Os09g38210) which were also downregulated in expression. Some auxin-responsive genes were downregulated in expression at 1dpi (LOC_Os03g58350, LOC_09g35870) and 14dpi (LOC_05g48270, LOC_01g67030, LOC_Os01g48850). Among early auxin response genes, the *SAUR* gene family is the largest and has been implicated in the regulation of a wide range of plant physiological and developmental processes [69]. At 1dpi, we identified the *SAUR* genes (e.g., LOC_Os01g56240, LOC_Os09g37460, LOC_Os06g50040, LOC_Os02g24700) to be differentially expressed in rice roots. Only one *SAUR* gene (LOC_Os06g50040) was induced in expression at 1dpi. The others were all downregulated in expression. Similarly, at 14dpi all the *SAUR* genes (e.g., LOC_Os06g04590, LOC_Os08g35110, LOC_Os02g05060, LOC_Os06g50040) were downregulated in expression. Another study reported that auxin-responsive genes were downregulated in expression during rice-*Herbaspirillum* interactions [8]. Therefore, it is tempting to speculate that the repression of plant-derived auxin pathways might be important for rice-*A*. *brasilense* interactions. Ethylene is another important plant hormone that plays vital roles in different aspects of plant biology including plant-microbe symbioses [2, 22]. The plant enzyme 1-aminocyclopropane-1carboxylate (ACC) oxidase catalyzes essential steps in the ethylene biosynthetic pathway. We identified several ACC oxidase genes that were differentially expressed at both time points. At 1dpi, one ACC oxidase gene (LOC_Os09g27750) was upregulated in expression, while other ACC oxidase genes (LOC_Os08g30080, LOC_Os05g05670, LOC_Os09g27820, LOC_Os02g53180) were downregulated in expression. Similarly, at 14dpi we identified one ACC oxidase gene (LOC_Os08g30100) to be upregulated in expression while other ACC oxidase genes (LOC_Os05g05670, LOC_03g64280, LOC_Os05g05680) were downregulated in expression. The general expression pattern of these genes suggests that ethylene synthesis might be repressed during rice-*A*. *brasilense* interactions. A positive regulator of the ethylene signaling pathway is the *Ethylene Insensitive 2* (*EIN2*) gene. Genetic studies have shown that the *Medicago* ortholog of Arabidopsis *EIN2* is a negative regulator of symbiotic and pathogenic microbial associations [70]. At 14dpi, we identified an *EIN2* gene (LOC_Os07g06130) that was differentially expressed. One study reported that *ein2* (*skl*) mutant in *Medicago truncatula* was hyper colonized by nitrogen-fixing endophyte *Klebsiella pneumoniae* 342, suggesting that ethylene acts as an inhibitor of the endophytic colonization process [71]. Whether this rice *EIN2* gene is required for associations with *A*. *brasilense* will require more studies.

**Fig 5:**
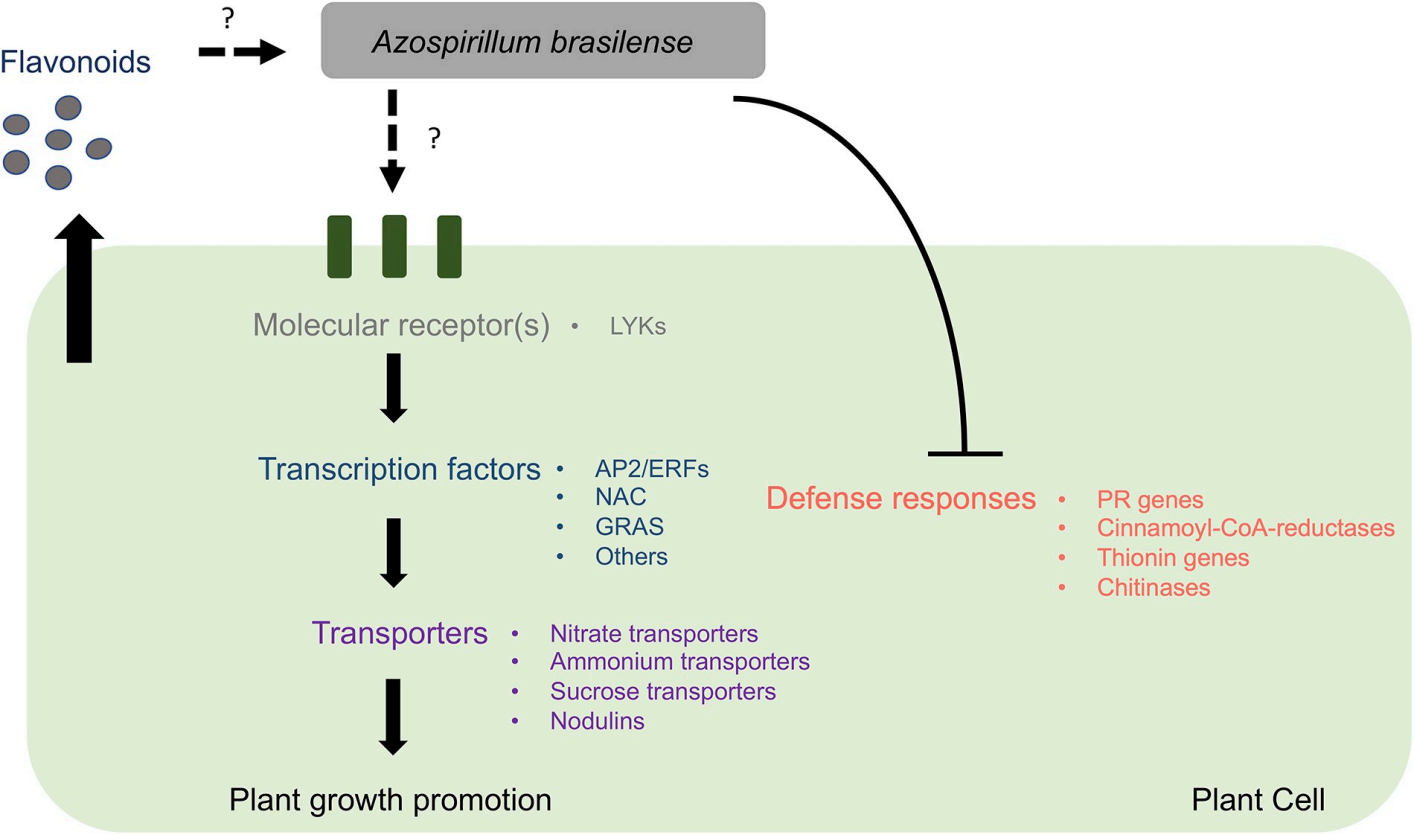
Overview of the putative molecular signaling pathway in plant roots during interactions with *Azospirillum brasilense*. The figure outlines a putative molecular signaling pathway in rice roots during interactions with *A*. *brasilense*. LYKs = LysM-RLK proteins with an active kinase domain, AP2/ERFs = APETALA 2/Ethylene response factors, NAC = NAM, ATAF1/2, and CUC, GRAS = GAI, RGA, and SCR, PR = pathogenesis-related.

To summarize, in this study we established an experimental system in which *A*. *brasilense* could colonize rice roots and promote plant growth. We observed similar effects in rice symbiotic mutants, *pollux* and *dmi3*, suggesting that these genes might not be required by the host plant to accommodate *A*. *brasilense*. Future studies should clarify the precise role of the common symbiotic pathway in these interactions. Using RNA-seq, we identified several excellent candidate genes which might be required for the rice-*A*. *brasilense* association. Our results suggest that the bacteria trigger a signaling pathway in the host plant roots that comprise a variety of protein kinases, transcription factors, and transporters culminating in plant growth promotion. Our data suggest the host defense responses are suppressed, as observed in other beneficial plant-microbe interactions(Fig 5; [17, 18, 26]). We also suggest that flavonoids might be involved in the initiation of this interaction. This dataset will serve as an excellent resource for improving our understanding of the interactions between non-legumes and beneficial bacteria. Most genetic studies on the host plant have been limited to legume-rhizobia and AM symbioses, but with advances in next-generation sequencing and genome-editing tools, we can now characterize other significant associations between non-legumes and beneficial bacteria. Identifying the genetic pathway(s) controlling these associations can have important implications for improving nitrogen fixation in non-legumes.

## Materials and methods

### Plant material and growth conditions

We used wild-type rice (*Oryza sativa* cv. Nipponbare) and *Tos17* insertion lines in *DMI3* (line NF8513) and in *POLLUX* (line NC6423) for the different experiments in this study [72, 73]. Seeds were surface sterilized and germinated as described in our previous study [74]. Germinated seedlings were transferred to 15-cm petri plates (#639102, Greiner bio-one, North Carolina, USA) containing low-N_2_ Fahraeus medium and allowed to grow for approximately 5–7 days in Percival growth chamber (#CU-22L, Iowa, USA) with 150 to 200 μmol m^-2^s^-1^ light intensity, and relative humidity of 65% before bacterial inoculation.

### Bacterial inoculation and bacterial counts

The bacterial inoculation of the rice roots was performed as described by Hiltenbrand et al [74]. Bacteria were grown on Tryptone Yeast-Extract (TY) media at 30°C to an optical density (600 nm) of 0.6 [75–77]. The cells were then resuspended in sterile water and used for inoculation. The control seedlings were treated with sterile water and the bacteria-treated seedlings were inoculated with 10^8^ cells/ml of *A*. *brasilense* and allowed to grow in the plant growth chamber as mentioned earlier. The root colonization was quantified as described by Hiltenbrand et al. (2016) with one minor modification. Here the seedlings were sampled six days post-inoculation with *A*. *brasilense*. The last wash was performed as mentioned in Hiltenbrand et al. (2016) to determine the efficiency of surface sterilization.

### RNA extraction and RNA sequencing

We extracted total RNA from the plant roots 1 and 14 days post bacterial inoculation using Qiagen RNeasy^®^ Plant Mini Kit (Cat #74904, California, USA) as described in Hiltenbrand *et al* [74]. We included three biological replicates for each sample. RNA quantification, library preparation, and sequencing were performed at the Research Technology Support Facility (RTSF), Michigan State University, East Lansing, MI, USA. Following RNA integrity check using a Bioanalyzer (Agilent Technologies), the sequencing libraries were prepared using the Illumina TruSeq Stranded mRNA Library Preparation Kit. Completed libraries were QC’d and quantified using a combination of Qubit dsDNA HS, Caliper LabChipGX HS DNA, and Kapa Illumina Library Quantification qPCR assays. All libraries were pooled in equimolar quantities and this pool was loaded on one lane of a HiSeq 4000 flow cell and sequenced in a 2×150bp paired-end format using HiSeq 4000 SBS reagents. Base calling was done by Illumina Real Time Analysis (RTA) v2.7.6 and output of RTA were demultiplexed and converted to FastQ format with Illumina Bcl2fastq v2.18.

### RNA sequencing data analysis

Raw paired-end reads were examined for a possible low base score, Illumina adapter and PCR contaminations using *fastQC*. Illumina TruSeq adapter sequences were detected in forward reads and Illumina Single End PCR Primer sequences were detected in reverse reads. We used *Trimmomatic* [78] to (1) remove Illumina TruSeq adapter and PCR primer sequences, (2) remove leading and trailing bases with low quality, (3) scan the read with a 4-base wide sliding window and cut when the average quality per base drops below 15, and (4) drop reads shorter than 36 bases long. S1 Table shows the summary of reads surviving these quality filtering criteria for day 1 and day 14 samples, respectively. Paired-end reads surviving the quality control criteria were processed using the Tophat-Cufflinks pipeline [79] to obtain normalized gene expression profiles. Paired-end reads were mapped to the rice genome (*Oryza sativa*) using *Tophat* (v2.0.12) [80], allowing two mismatches. The genome contigs (file *all*.*chrs*.*con*), gene annotations (file *all*.*gff3*) for 55986 loci, and short descriptions (file *all*.*locus_brief_info*.*7*.*man*) were downloaded from the Rice Genome Annotation Project [81]. Reads that align to annotated loci were quantified and normalized (FPKM normalized values) using *cufflinks* (v2.2.1) [82]. Differential expression (DE) analysis was performed using *cuffdiff* (part of the *Cufflinks* suite) and significant DE genes were defined as those with false discovery rate (FDR) <0.05 and absolute fold-change (FC) >2.

### Reverse-transcriptase PCR

The RNA-seq results were validated with select genes via reverse transcriptase PCR (RT-PCR) as described in [83]. Prior to cDNA synthesis, RNA samples were treated with the Ambion^®^ DNA-free^™^ DNase Treatment and Removal kit (Cat #AM1906, California, USA). We synthesized first strand cDNA from 300 ng of RNA using Thermo Scientific RevertAid RT Kit (Cat #K1691, Delaware, USA) using Oligo(dT)_18_ primers per manufacturer’s instructions. For the internal reference gene, we used *Cyclophilin* in our RT-PCR analysis [74, 84]. The table of genes and their corresponding primer sequences are listed in S7 Table.

## Supporting information

S1 Table Sequence data summary. (XLSX)

S2 Table List of differentially expressed genes in rice roots at 1dpi with *A*. *brasilense*. (XLSX)

S3 Table List of differentially expressed genes in rice roots at 14dpi with *A*. *brasilense*. (XLSX)

S4 Table List of differentially expressed transcription factors. (XLSX)

S5 Table List of differentially expressed protein kinases. (XLSX)

S6 Table List of differentially expressed transporters. (XLSX)

S7 Table List of primers used in RT-PCR. (XLSX)
